# Li_3_Al(MoO_2_)_2_O_2_(AsO_4_)_2_

**DOI:** 10.1107/S160053680900631X

**Published:** 2009-02-25

**Authors:** Mounir Hajji, Mohamed Faouzi Zid, Ahmed Driss

**Affiliations:** a Laboratoire de Matériaux et Cristallochimie, Faculté des Sciences, Université de Tunis ElManar, 2092 ElManar Tunis, Tunisie

## Abstract

Single crystals of trilithium(I) aluminium(III) bis­[dioxidomolybdenum(VI)] dioxide bis­[arsenate(V)], Li_3_AlMo_2_As_2_O_14_, have been prepared by solid-state reaction at 788 K. The structure consists of AsO_4_ tetra­hedra, AlO_6_ octa­hedra and Mo_2_O_10_ groups sharing corners to form a three-dimensional framework containing channels running respectively along the [100] and [010] directions, where the Li^+^ ions are located. This structure is compared with compounds having (*MX*_2_O_12_)_*n*_ chains (*M* = Mo, Al and *X* = P, As) and others containing *M*_2_O_10_ (*M* = Mo, Fe) dimers.

## Littérature assocíee

Pour le détail de la préparation, voir: Hajji *et al.* (2004 ▶, 2005 ▶); Zid *et al.* (1997 ▶, 1998 ▶). Pour le détail des composés avec structures reliées, voir: Lii *et al.* (1989 ▶); Leclaire *et al.* (1990 ▶); Driss & Jouini (1989 ▶); Guesdon *et al.* (1994 ▶); Borel *et al.* (1994 ▶); LeBail *et al.* (1995 ▶). Pour le détail des propriétés des composés reliés, voir: Manthiram & Goodenough, 1989 ▶; Tarascon *et al.*, 1991 ▶; Sigala *et al.*, 1997 ▶; Padhi *et al.*, 1997 ▶; Masquelier *et al.*, 1998 ▶. Pour le calcul des valences des liaisons, voir: Brown & Altermatt (1985 ▶)

## Partie expérimentale

### 

#### Données crystallines

Li_3_Al(MoO_2_)_2_O_2_(AsO_4_)_2_
*M* *_r_* = 613.52
Triclinique, 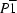

*a* = 5.213 (1) Å
*b* = 5.426 (1) Å
*c* = 9.474 (2) Å
α = 95.98 (2)°
β = 102.25 (1)°
γ = 105.30 (1)°
*V* = 248.92 (9) Å^3^
*Z* = 1
Mo *K*α radiation
μ = 9.29 mm^−1^
*T* = 298 K
0.20 × 0.15 × 0.12 mm

#### Collection des données

Diffractomètre Enraf–Nonius TurboCAD-4
Correction d’absorption: ψ scan (North *et al.*, 1968 ▶) *T*_min_ = 0.209, *T*_max_ = 0.328
1663 réflexions mesurées
1084 réflexions independantes
1077 réflexions avec *I* > 2σ(*I*)
*R*_int_ = 0.007
2 réflexions de référence fréquence: 120 min décroissance d’intensité: 1.1%

#### Affinement

*R*[*F* ^2^ > 2σ(*F* ^2^)] = 0.013
*wR*(*F* ^2^) = 0.039
*S* = 1.06
1084 réflexions
104 paramètres
Δρ_max_ = 0.64 e Å^−3^
Δρ_min_ = −0.41 e Å^−3^

### 

Collection des données: *CAD-4 EXPRESS* (Duisenberg, 1992 ▶; Macíček & Yordanov, 1992 ▶); affinement des paramètres de la maille: *CAD-4 EXPRESS*; reduction des données: *XCAD4* (Harms & Wocadlo, 1995 ▶); programme(s) pour la solution de la structure: *SHELXS97* (Sheldrick, 2008 ▶); programme(s) pour l’affinement de la structure: *SHELXL97* (Sheldrick, 2008 ▶); graphisme moléculaire: *DIAMOND* (Brandenburg, 1998 ▶); logiciel utilisé pour préparer le matériel pour publication: *WinGX* (Farrugia, 1999 ▶).

## Supplementary Material

Crystal structure: contains datablocks I, global. DOI: 10.1107/S160053680900631X/br2097sup1.cif

Structure factors: contains datablocks I. DOI: 10.1107/S160053680900631X/br2097Isup2.hkl

Additional supplementary materials:  crystallographic information; 3D view; checkCIF report

## Figures and Tables

**Fig. 1.**
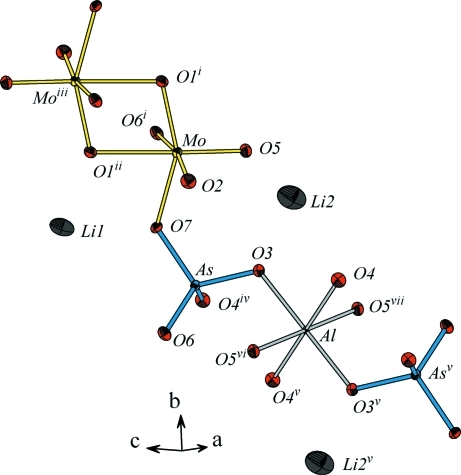
Unité structurale dans Li3AlMo2As2O14. Les ellipsoïdes d'agitation thermique ont 50% de probabilité. [Codes de symétrie: (i) x, y + 1, z; (ii) -x + 1, -y + 1, -z + 1; (iii) -x + 1, -y + 2, -z + 1; (iv) x - 1, y, z; (v) -x + 1, -y, -z; (vi) x, y - 1, z; (vii) -x + 1, -y + 1, -z.]

**Fig. 2.**
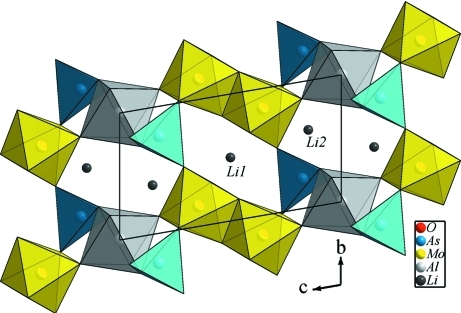
Projection de la structure de Li3AlMo2As2O14 selon a montrant les deux types de canaux où logent les cations Li+.

**Fig. 3.**
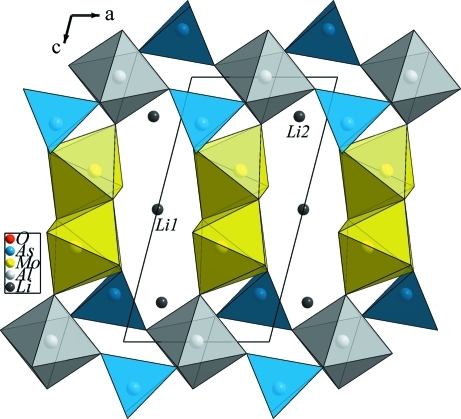
Projection de la structure de Li3AlMo2As2O14 selon b montrant les larges canaux où résident les cations Li+.

## supplementary crystallographic information

### Comment

Les oxydes mixtes au lithium présentent un intérêt pratique potentiel du fait de leurs utilisations comme électrolytes solides ou électrodes positives dans les batteries au lithium (Manthiram & Goodenough, 1989; Tarascon *et al.*, 1991; Sigala *et al.*, 1997; Padhi *et al.*, 1997; Masquelier *et al.*, 1998). Dans ce cadre, nous avons entrepris l'exploration du diagramme Li–Mo–As–O dans lequel nous avons précédemment caractérisé les composés β-LiMoO_2_AsO_4_ (Hajji *et al.*, 2004) et Li(MoO_2_)_2_OAsO_4_ (Hajji *et al.*, 2005). Un nouvel oxyde mixte de formulation Li_3_AlMo_2_As_2_O_14_ a été également obtenu par réaction à l'état solide au cours de la synthèse de la phase visée Li_2_MoO_2_As_2_O_7_ de formulation analogue à K_2_MoO_2_As_2_O_7_ (Zid *et al.*, 1997) ou bien Rb_2_MoO_2_As_2_O_7_ (Zid *et al.*, 1998) ayant des structures à charpentes anioniques bidimensionnelles.

L'unité asymétrique dans le composé Li_3_AlMo_2_As_2_O_14_ est constituée de deux octaèdres MoO_6_ reliés par une arête, de deux tétraèdres AsO_4_ et d'un octaèdre AlO_6_ partageant des sommets (Fig. 1). La structure peut être décrite à partir de chaînes infinies (AlAs_2_O_12_), parallèles à la direction [100], reliées entre elles par des bioctaèdres Mo_2_O_10_ pour former une charpente tridimensionnelle délimitant des tunnels, disposés respectivement selon *a* (Fig. 2) et suivant la direction *b* (Fig. 3), où résident les cations Li^+^. Le calcul des différentes valences des liaisons utilisant la formule empirique de Brown (Brown & Altermatt, 1985) vérifie bien les valeurs de charges des ions Mo (5,95), As (4,88), Al (3,02) et Li (1,05) dans la phase étudiée.

La comparaison de la structure du composé étudié avec celles des travaux antérieurs montre quelques points communs. En effet, les chaînes de type (*MX*_2_O_12_)*_n_* sont rencontrées dans les composés de formulation *AM*_2_(*X*_2_O_7_)_2_ (*M* = Mo, Al; *X* = P, As; *A* = Na et K) (Lii *et al.*, 1989; Leclaire *et al.*, 1990; Driss & Jouini, 1989). Dans ces derniers un octaèdre MO_6_ partage ses sommets avec quatre groupements *X*_2_O_7_. Par contre, il met en commun dans la phase étudiée, ses six sommets avec seulement quatre unités AsMoO_9_ différentes. Cette différence est essentiellement due au remplacement d'un tétraèdre *X*O_4_ par un octaèdre MoO_6_. De même, les groupements *M*_2_O_10_ sont rencontrés dans les composés de formulation *A*Mo_3_P_2_O_14_ (*A* = Na, K) sous forme de Mo_2_O_10_ (Guesdon *et al.*, 1994; Borel *et al.*, 1994) ou bien dans la structure du FeVMoO_7_ sous forme de Fe_2_O_10_ (LeBail *et al.*, 1995). Ces derniers phosphates présentent des structures bidimensionnelles dont les groupements Mo_2_O_10_ partagent leurs sommets avec les polyèdres PO_4_ et MoO_6_. Dans le composé FeVMoO_7_, chaque dimère Fe_2_O_10_ partage ses dix sommets avec quatre tétraèdres MoO_4_ et six tétraèdres VO_4_. Une différence nette est donc observée dans le comportement des dimères *M*_2_O_10_ rencontrés dans notre structure et celles de la bibliographie. En effet, chaque groupement Mo_2_O_10_ dans la charpente de Li_3_AlMo_2_As_2_O_14_ ne partage ses sommets qu'avec seulement deux tétraèdres AsO_4_ et deux octaèdres AlO_6_ et conduit, contrairement aux autres composés, à une structure très ouverte. Cette caractéristique structurale, ainsi que la disposition des cations Li^+^ dans des larges canaux (Fig. 2 et 3), favorisent la mobilité de ces ions Li^+^ et laisse présager la propriété de conductivité ionique pour le composé Li_3_AlMo_2_As_2_O_14_. Afin d'utiliser ces données structurales et les relier aux propriétés physico-chimiques, en particulier de conduction ionique, et dès l'obtention d'une phase polycristalline pure du Li_3_AlMo_2_As_2_O_14_, des mesures électriques moyennant un pont d'impédance complexe de type HP4192A seront réalisées.

### Experimental

La stoechiométrie du mélange réactionnel est destinée en premier à la synthèse du composé Li_2_MoAs_2_O_9_. Les réactifs de départ NH_4_H_2_AsO_4_ (préparé au laboratoire, ASTM 01-0775), (NH_4_)_2_Mo_4_O_13_ (Fluka, 85%), Li_2_CO_3_ (Fluka, 99%) ont été pris dans les proportions Li:Mo:As = 2:1:2. Le mélange, finement broyé, a été mis dans un creuset en porcelaine, placé dans un four puis préchauffé à l'air à 623 K pendant 24 heures en vue d'éliminer les composés volatils. Le résidu final est porté à la fusion (813 K) par accident, où une attaque du creuset en porcelaine a eu lieu, puis refroidi lentement jusqu'à 778 K. Il est abondonné à cette température durant trois jours pour favoriser la cristallisation. Le résidu final a subi en premier un refroidissement lent (5°/h) jusqu'à 738 K puis un second rapide (50°/h) jusqu'à la température ambiante. Quelques cristaux jaunâtres, de taille suffisante pour les mesures des intensités, ont été séparés du flux par l'eau bouillante. L'analyse des données de diffraction des rayons-X sur monocristal, les paramètres géométriques ainsi que la qualité des résultats trouvés confirment la présence de l'aluminium dans la structure. Cet élément est probablement diffusé dans le cristal choisi, à partir du creuset en porcelaine.

### Crystal data

**Table:**

Li_3_Al(MoO_2_)_2_O_2_(AsO_4_)_2_	*Z* = 1
*M**_r_* = 613.52	*F*(000) = 284
Triclinic, *P*1	*D*_x_ = 4.093 Mg m^−^^3^
Hall symbol: -P 1	Mo *K*α radiation, λ = 0.71073 Å
*a* = 5.213 (1) Å	Cell parameters from 25 reflections
*b* = 5.426 (1) Å	θ = 10–16°
*c* = 9.474 (2) Å	µ = 9.29 mm^−^^1^
α = 95.98 (2)°	*T* = 298 K
β = 102.25 (1)°	Prism, yellow
γ = 105.30 (1)°	0.20 × 0.15 × 0.12 mm
*V* = 248.92 (9) Å^3^	

### Data collection

**Table:**

Enraf–Nonius TurboCAD-4 diffractometer	1077 reflections with *I* > 2σ(*I*)
Radiation source: fine-focus sealed tube	*R*_int_ = 0.007
graphite	θ_max_ = 27.0°, θ_min_ = 2.2°
ω/2θ scans	*h* = −2→6
Absorption correction: ψ scan (North *et al.*, 1968)	*k* = −6→6
*T*_min_ = 0.209, *T*_max_ = 0.328	*l* = −12→12
1663 measured reflections	2 standard reflections every 120 min
1084 independent reflections	intensity decay: 1.1%

### Refinement

**Table:**

Refinement on *F*^2^	Primary atom site location: structure-invariant direct methods
Least-squares matrix: full	Secondary atom site location: difference Fourier map
*R*[*F*^2^ > 2σ(*F*^2^)] = 0.013	*w* = 1/[σ^2^(*F*_o_^2^) + (0.021*P*)^2^ + 0.6735*P*] where *P* = (*F*_o_^2^ + 2*F*_c_^2^)/3
*wR*(*F*^2^) = 0.039	(Δ/σ)_max_ = 0.001
*S* = 1.06	Δρ_max_ = 0.64 e Å^−^^3^
1084 reflections	Δρ_min_ = −0.41 e Å^−^^3^
104 parameters	Extinction correction: *SHELXL97* (Sheldrick, 2008), Fc^*^=kFc[1+0.001xFc^2^λ^3^/sin(2θ)]^-1/4^
0 restraints	Extinction coefficient: 0.0263 (14)

### Special details

**Table:**

Geometry. All e.s.d.'s (except the e.s.d. in the dihedral angle between two l.s. planes) are estimated using the full covariance matrix. The cell e.s.d.'s are taken into account individually in the estimation of e.s.d.'s in distances, angles and torsion angles; correlations between e.s.d.'s in cell parameters are only used when they are defined by crystal symmetry. An approximate (isotropic) treatment of cell e.s.d.'s is used for estimating e.s.d.'s involving l.s. planes.
Refinement. Refinement of *F*^2^ against ALL reflections. The weighted *R*-factor *wR* and goodness of fit *S* are based on *F*^2^, conventional *R*-factors *R* are based on *F*, with *F* set to zero for negative *F*^2^. The threshold expression of *F*^2^ > σ(*F*^2^) is used only for calculating *R*-factors(gt) *etc*. and is not relevant to the choice of reflections for refinement. *R*-factors based on *F*^2^ are statistically about twice as large as those based on *F*, and *R*- factors based on ALL data will be even larger.

### Fractional atomic coordinates and isotropic or equivalent isotropic displacement parameters (Å^2^)

**Table:**

	*x*	*y*	*z*	*U*_iso_*/*U*_eq_	
Mo	0.49876 (4)	0.84099 (4)	0.35495 (2)	0.00633 (9)	
As	0.15020 (5)	0.23501 (5)	0.17692 (3)	0.00601 (10)	
Al	0.5000	0.0000	0.0000	0.0026 (2)	
Li1	0.0000	0.5000	0.5000	0.0225 (16)	
Li2	0.8077 (14)	0.6121 (14)	0.1486 (9)	0.0392 (16)	
O1	0.6756 (4)	0.1635 (4)	0.4618 (2)	0.0099 (4)	
O2	0.7195 (4)	0.6684 (4)	0.4132 (2)	0.0132 (4)	
O3	0.4158 (4)	0.2923 (4)	0.0959 (2)	0.0109 (4)	
O4	0.8654 (4)	0.2067 (4)	0.0441 (2)	0.0110 (4)	
O5	0.5596 (4)	0.8659 (4)	0.1781 (2)	0.0098 (4)	
O6	0.1363 (4)	−0.0156 (4)	0.2672 (2)	0.0101 (4)	
O7	0.1633 (4)	0.5095 (4)	0.2895 (2)	0.0106 (4)	

### Atomic displacement parameters (Å^2^)

**Table:**

	*U*^11^	*U*^22^	*U*^33^	*U*^12^	*U*^13^	*U*^23^
Mo	0.00695 (13)	0.00664 (13)	0.00518 (13)	0.00175 (8)	0.00146 (8)	0.00074 (8)
As	0.00626 (14)	0.00629 (14)	0.00524 (14)	0.00179 (10)	0.00123 (10)	0.00057 (9)
Al	0.0027 (5)	0.0038 (4)	0.0011 (4)	0.0005 (4)	0.0004 (3)	0.0002 (3)
Li1	0.018 (4)	0.018 (4)	0.031 (4)	0.007 (3)	−0.001 (3)	0.011 (3)
Li2	0.031 (3)	0.031 (3)	0.063 (5)	0.017 (3)	0.014 (3)	0.017 (3)
O1	0.0108 (9)	0.0086 (9)	0.0086 (8)	0.0001 (7)	0.0024 (7)	0.0012 (7)
O2	0.0137 (10)	0.0127 (9)	0.0146 (10)	0.0055 (8)	0.0035 (8)	0.0044 (8)
O3	0.0115 (9)	0.0094 (9)	0.0126 (9)	0.0020 (7)	0.0067 (7)	0.0010 (7)
O4	0.0086 (9)	0.0135 (9)	0.0092 (9)	0.0025 (7)	−0.0009 (7)	0.0025 (7)
O5	0.0093 (9)	0.0115 (9)	0.0083 (9)	0.0020 (7)	0.0025 (7)	0.0020 (7)
O6	0.0099 (9)	0.0110 (9)	0.0114 (9)	0.0044 (7)	0.0032 (7)	0.0061 (7)
O7	0.0109 (9)	0.0086 (9)	0.0108 (9)	0.0004 (7)	0.0048 (7)	−0.0023 (7)

### Geometric parameters (Å, °)

**Table:**

Mo—O2	1.707 (2)	Al—O3^v^	1.942 (2)
Mo—O5	1.782 (2)	Al—O3	1.942 (2)
Mo—O1^i^	1.822 (2)	Li1—O2^ii^	2.000 (2)
Mo—O7	2.080 (2)	Li1—O2^iv^	2.000 (2)
Mo—O1^ii^	2.123 (2)	Li1—O1^ii^	2.073 (2)
Mo—O6^i^	2.261 (2)	Li1—O1^iv^	2.073 (2)
Mo—Mo^iii^	3.0849 (8)	Li1—O7^viii^	2.328 (2)
As—O6	1.6737 (19)	Li1—O7	2.328 (2)
As—O4^iv^	1.6903 (19)	Li2—O5	2.159 (7)
As—O3	1.6960 (19)	Li2—O3	2.225 (7)
As—O7	1.7164 (19)	Li2—O6^ix^	2.272 (8)
Al—O4^v^	1.870 (2)	Li2—O7^x^	2.275 (7)
Al—O4	1.870 (2)	Li2—O4	2.435 (7)
Al—O5^vi^	1.905 (2)	Li2—O3^vii^	2.533 (8)
Al—O5^vii^	1.905 (2)	Li2—O2	2.645 (8)
			
O2—Mo—O5	98.47 (9)	O2^ii^—Li1—O2^iv^	180.000 (1)
O2—Mo—O1^i^	102.62 (9)	O2^ii^—Li1—O1^ii^	86.18 (8)
O5—Mo—O1^i^	104.19 (9)	O2^iv^—Li1—O1^ii^	93.82 (8)
O2—Mo—O7	92.53 (9)	O2^ii^—Li1—O1^iv^	93.82 (8)
O5—Mo—O7	96.70 (8)	O2^iv^—Li1—O1^iv^	86.18 (8)
O1^i^—Mo—O7	151.88 (8)	O1^ii^—Li1—O1^iv^	180.0
O2—Mo—O1^ii^	96.79 (9)	O2^ii^—Li1—O7^viii^	90.24 (8)
O5—Mo—O1^ii^	163.86 (8)	O2^iv^—Li1—O7^viii^	89.76 (8)
O1^i^—Mo—O1^ii^	77.39 (9)	O1^ii^—Li1—O7^viii^	106.95 (7)
O7—Mo—O1^ii^	77.39 (7)	O1^iv^—Li1—O7^viii^	73.05 (7)
O2—Mo—O6^i^	167.49 (8)	O2^ii^—Li1—O7	89.76 (8)
O5—Mo—O6^i^	83.83 (8)	O2^iv^—Li1—O7	90.24 (7)
O1^i^—Mo—O6^i^	88.62 (8)	O1^ii^—Li1—O7	73.05 (7)
O7—Mo—O6^i^	74.97 (8)	O1^iv^—Li1—O7	106.95 (7)
O1^ii^—Mo—O6^i^	80.14 (7)	O7^viii^—Li1—O7	180.0
O6—As—O4^iv^	115.31 (9)	O5—Li2—O3	85.6 (3)
O6—As—O3	112.28 (9)	O5—Li2—O6^ix^	79.0 (2)
O4^iv^—As—O3	106.27 (9)	O3—Li2—O6^ix^	158.9 (4)
O6—As—O7	111.28 (10)	O5—Li2—O7^x^	136.7 (4)
O4^iv^—As—O7	100.31 (10)	O3—Li2—O7^x^	112.7 (3)
O3—As—O7	110.70 (9)	O6^ix^—Li2—O7^x^	71.1 (2)
O4^v^—Al—O4	180.00 (15)	O5—Li2—O4	151.9 (4)
O4^v^—Al—O5^vi^	88.07 (8)	O3—Li2—O4	68.8 (2)
O4—Al—O5^vi^	91.93 (8)	O6^ix^—Li2—O4	128.6 (3)
O4^v^—Al—O5^vii^	91.93 (8)	O7^x^—Li2—O4	67.36 (19)
O4—Al—O5^vii^	88.07 (8)	O5—Li2—O3^vii^	69.4 (2)
O5^vi^—Al—O5^vii^	180.00 (10)	O3—Li2—O3^vii^	81.2 (3)
O4^v^—Al—O3^v^	87.61 (9)	O6^ix^—Li2—O3^vii^	106.3 (3)
O4—Al—O3^v^	92.39 (9)	O7^x^—Li2—O3^vii^	148.8 (4)
O5^vi^—Al—O3^v^	88.69 (8)	O4—Li2—O3^vii^	94.6 (3)
O5^vii^—Al—O3^v^	91.31 (8)	O5—Li2—O2	65.9 (2)
O4^v^—Al—O3	92.39 (9)	O3—Li2—O2	84.0 (2)
O4—Al—O3	87.61 (9)	O6^ix^—Li2—O2	76.6 (3)
O5^vi^—Al—O3	91.31 (8)	O7^x^—Li2—O2	77.0 (2)
O5^vii^—Al—O3	88.69 (8)	O4—Li2—O2	120.1 (3)
O3^v^—Al—O3	180.00 (12)	O3^vii^—Li2—O2	133.7 (3)

Symmetry codes: (i) *x*, *y*+1, *z*; (ii) −*x*+1, −*y*+1, −*z*+1; (iii) −*x*+1, −*y*+2, −*z*+1; (iv) *x*−1, *y*, *z*; (v) −*x*+1, −*y*, −*z*; (vi) *x*, *y*−1, *z*; (vii) −*x*+1, −*y*+1, −*z*; (viii) −*x*, −*y*+1, −*z*+1; (ix) *x*+1, *y*+1, *z*; (x) *x*+1, *y*, *z*.
